# Supplementary material for: Amphiphilic Poly(N-vinylpyrrolidone) Nanoparticles Conjugated with DR5-Specific Antitumor Cytokine DR5-B for Targeted Delivery to Cancer Cells
Source: Pharmaceutics. 2021 Sep 7;13(9):1413. doi: 10.3390/pharmaceutics13091413 (PMC8464842; doi:10.3390/pharmaceutics13091413)
Supplement: Supplementary file 1 [file pharmaceutics-13-01413-s001.zip › pharmaceutics-1343028-supplementary.pdf]

pharmaceutics

# Supplementary Materials: Amphiphilic Poly(*N*-Vinylyrrolidone) Nanoparticles Conjugated with DR5-Specific Antitumor Cytokine DR5-B for Targeted Delivery to Cancer Cells

**Anne Yagolovich, Andrey Kuskov, Pavel Kulikov, Leily Kurbanova, Dmitry Bagrov, Artem Artykov, Marine Gasparian, Svetlana Sizova, Vladimir Oleinikov, Anastasia Gileva, Mikhail Kirpichnikov, Dmitry Dolgikh and Elena Markvicheva**

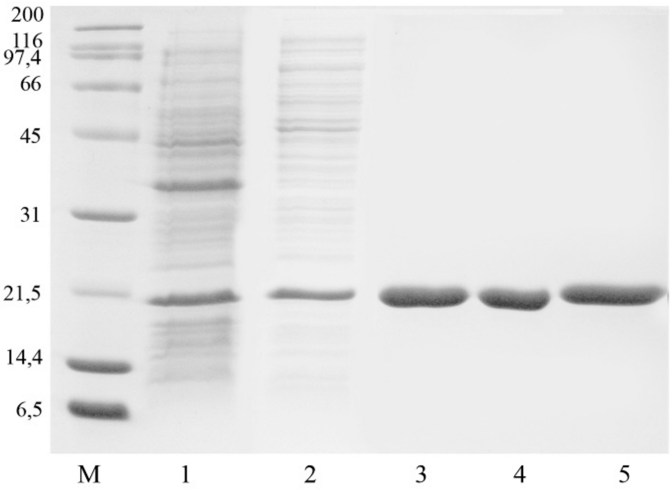


**Figure S1.** Expression and purification of DR5-B/V114C protein. Samples were analyzed in 14% Tris-glycine SDS-PAGE. Lanes: M–molecular weight markers; 1–20 µL of cell culture after expression; 2-soluble cell fraction; 3–5–purified protein DR5-B/V114C.

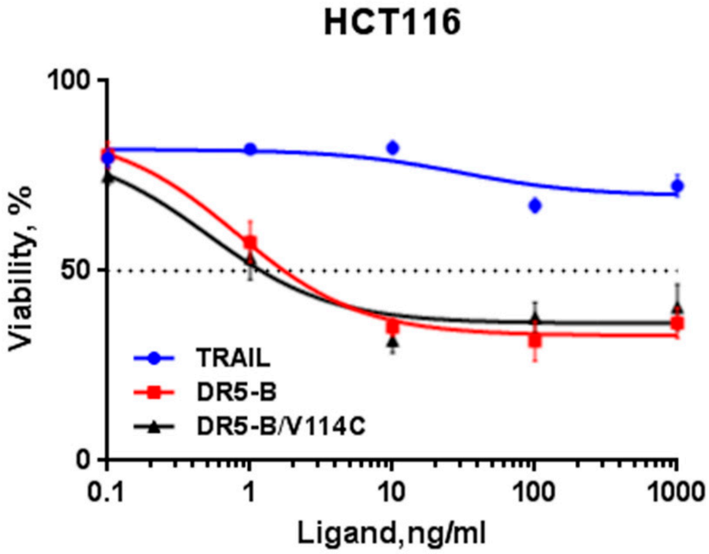


**Figure S2.** DR5-B/V114C cytotoxic activity in human colorectal carcinoma HCT116 cell line. Cells were treated with ligands at the indicated concentrations for 24 h. Cell viability was determined by MTT test. Mean ± Standard Deviation ($n$ = 3).

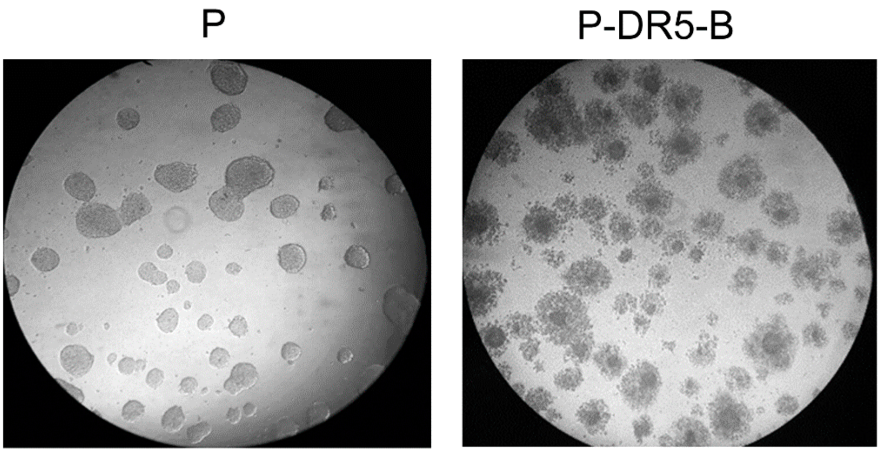


**Figure S3.** Morphology of 3D tumor spheroids of HCT116 colorectal carcinoma cells after 24 h incubation with P-DR5-B at concentration corresponding to 100 ng/mL of the DR5-B ligand.

**Table S1.** The values of standard errors of the regression (S) for IC50 of free DR5-B and the P-DR5-B nanoparticles in 2D and 3D in vitro models.

| | **2D MCF-7** | | **3D MCF-7** | | **3D HCT116** | | **3D HT29** | |
|---|---|---|---|---|---|---|---|---|
| Incubation time, h | 24 | 48 | 24 | 48 | 24 | 48 | 24 | 48 |
| DR5-B | 0.3577 | 0.4950 | 0.1552 | 0.1253 | 1.135 | 0.6893 | 0.2881 | 4.431 |
| P-DR5-B | 0.1202 | 0.2405 | 0.1937 | 0.1046 | 0.7644 | 0.4700 | 0.1712 | 0.2225 |
